# Fully printable, strain-engineered electronic wrap for customizable soft electronics

**DOI:** 10.1038/srep45328

**Published:** 2017-03-24

**Authors:** Junghwan Byun, Byeongmoon Lee, Eunho Oh, Hyunjong Kim, Sangwoo Kim, Seunghwan Lee, Yongtaek Hong

**Affiliations:** 1 Department of Electrical and Computer Engineering, Inter-University Semiconductor Research Center (ISRC), Seoul National University, 1 Gwanak-ro, Gwanak-gu, Seoul 151-744, Korea

## Abstract

Rapid growth of stretchable electronics stimulates broad uses in multidisciplinary fields as well as industrial applications. However, existing technologies are unsuitable for implementing versatile applications involving adaptable system design and functions in a cost/time-effective way because of vacuum-conditioned, lithographically-predefined processes. Here, we present a methodology for a fully printable, strain-engineered electronic wrap as a universal strategy which makes it more feasible to implement various stretchable electronic systems with customizable layouts and functions. The key aspects involve inkjet-printed rigid island (PRI)-based stretchable platform technology and corresponding printing-based automated electronic functionalization methodology, the combination of which provides fully printed, customized layouts of stretchable electronic systems with simplified process. Specifically, well-controlled contact line pinning effect of printed polymer solution enables the formation of PRIs with tunable thickness; and surface strain analysis on those PRIs leads to the optimized stability and device-to-island fill factor of strain-engineered electronic wraps. Moreover, core techniques of image-based automated pinpointing, surface-mountable device based electronic functionalizing, and one-step interconnection networking of PRIs enable customized circuit design and adaptable functionalities. To exhibit the universality of our approach, multiple types of practical applications ranging from self-computable digital logics to display and sensor system are demonstrated on skin in a customized form.

Rapid advances in stretchable electronics have significantly extended the device functionality and potential applications in ways that allow intimate interfaces between electronic systems and unconventional, soft surfaces. In conjunction with advanced material assemblies^1^,^2^,^3^,^4^, well-designed architectures^5^,^6^,^7^,^8^, and theoretical backgrounds^9^,^10^, electronic units nowadays can be attached to human skin and functionally operated under skin-like deformation without any electrical or mechanical failure, providing a new paradigm of electronics in a wearable, skin-conformable form. Promising technologies have been developed to enable a new class of applications such as imperceptible, long-term monitoring of individual’s activity^11^,^12^,^13^,^14^,^15^, noninvasive control/measurement of clinical information such as blood^16^,^17^, perspiration^18^,^19^, and even brain^20^, and motion-activated user-interfaces^1^,^21^ in the form of electronic epidermis. The forefront of such technologies includes ultrathin patterned silicon devices^5^,^6^,^7^,^11^,^13^, imperceptible electronic foils^12^,^17^,^22^,^23^, and rigid island-embedded electronic systems^14^,^15^,^23^. Along with these technical improvements and pioneering works, a fundamental framework for versatile uses of stretchable electronics in multi-, cross-disciplinary fields starts to be established.

Despite these achievements, however, existing options in sophisticated, inadaptable methodologies that were mainly based on multistep, lithographically-predefined process have confined design freedom, system function, and categorical usage in a predefined way. Furthermore, limitations in scalability, facile processability, and cost efficiency of such methodologies have precluded large-area, rapid implementation; thereby disrupting broad, adaptable uses of such state-of-the-art electronic systems. In other words, stretchable electronic systems with adaptable design and utilization―namely, “customizable electronics”― have remained distant prospect compared to rigid electronics where customizable circuit layouts and corresponding functions are easily integrated in terms of “electronic design automation” in widely-known printed circuit board (PCB) technology^24^.

One way to meet the requirements for those limitations is to address a direct drop-on-demand (DOD) printing process. Due to the feasibility of scalable, arbitrary patterning with minimized material waste, such DOD printing process can further diversify the design freedom and involved functionality of stretchable electronic systems both while simplifying process steps (by skipping vacuum and photolithography processes) and minimizing cost. Moreover, together with well-developed substrate engineering technology that provides locally strain-free areas among the soft matrix for *in situ* protecting active and/or deformation-sensitive parts^25^,^26^,^27^,^28^,^29^,^30^,^31^,^32^, the direct printing method can create a great synergy: if one-step printing of stretchable conductors forms multi-configured, customized interconnection networks between spatially distributed electronic units supported on rigid islands, adaptable functionalities would be easily achieved in conjunction with the customized circuit design.

Here, we present a methodology for a fully printable, strain-engineered electronic wrap as a key enabling platform for the true meaning of customizable electronics on skin―where multi-purpose, multi-configuration, and versatile functions can be addressed. The strain-engineered electronic wrap concept has the following two key aspects:

1. inkjet-printed rigid island (PRI)-based stretchable soft platform
2. printing-based automated electronic functionalization

As a soft substrate engineering technology, the methodology to form multilayered PRI structure and corresponding PRI-embedded soft wrap (PRISW) (thickness ~50 μm) is developed. In particular, well-controlled contact line pinning effect of printed polymer solution enables the formation of PRIs with tunable thickness; offering diversity in structural parameters of PRIs just in a single batch. Surface strain analysis on those PRISWs with multiple types of PRIs leads to the optimized stability, improved skin-conformability, device-to-island fill factor, and device density of the system (Supplementary Table S1). As the second key aspect, printing-based automated electronic functionalization technology can easily convert an arbitrary PRISW into the functional electronic wrap. Specifically, core techniques of (i) image-based automated pinpointing, (ii) surface-mountable device (SMD) based electronic functionalizing, and (iii) one-step interconnection networking of PRIs enable customized circuit design and adaptable functionalities within a short period of process time.

## Results

### Concept of strain-engineered soft electronic wrap

**Figure 1:**
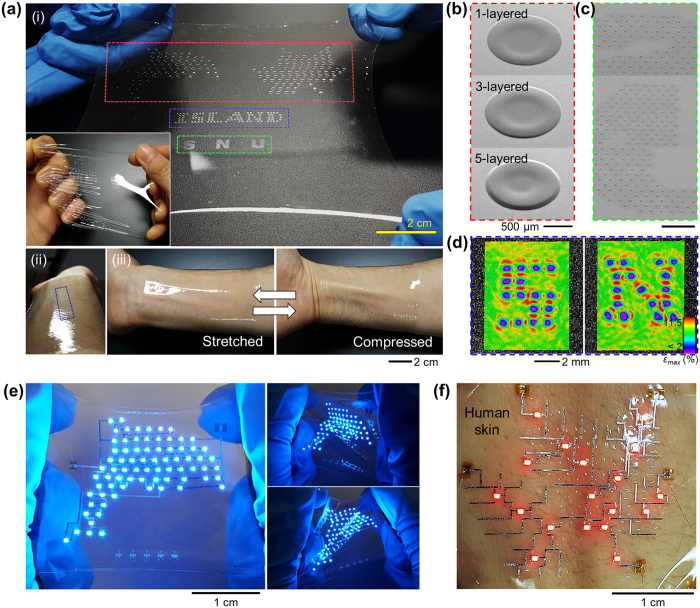
Concept of fully-printable, strain-engineered, customized electronic wrap. (**a**) Photograph of a soft strain-engineered wrap that contains multi-scale/multi-structured inkjet-printed rigid islands (PRIs) in a customized configuration (dolphin, maple leaf, letters) (i). Each PRI subsection either a dolphin or a maple leaf can separately be obtained by a physical cutting for an individual use. Owing to the transparency, PRIs are perceived only if illuminated by the tilted light incidence (ii). The wrap shows superb stretchability and conformability (iii). (**b**) Scanning electron microscope (SEM) images of multistep-printed PRIs (diameter ~1450 μm) constructing the “dolphin” and “maple leaf”. (**c**) SEM images of micro-size PRIs (diameter ~80 μm) constructing the letter “SNU”. (d) Digital image correlation (DIC) results representing strain-free areas (<2%) in a programmed configuration under the biaxial strain of *ε*_*x*_ = *ε*_*y*_ = 10%, constructing the letter “ISLAND” (Supplementary Fig. S4). (**e**) Photographs of fully-printed, customized electronic wrap display embodying a dolphin. The electronic wrap display is stretchable and twistable. (**f**) Photographs of fully-printed, customized electronic wrap display embodying a maple leaf. The system is attached to human skin, serving as a wrap-like electronic skin. Detailed branches of the maple leaf are described by customized interconnection networks in combination with selected PRIs.

Figure 1 briefly summarizes our approach of fully printable (from substrate engineering to electronic functionalization of the soft wrap), customized electronic wrap. Inspired by the stretchable bead network connected by springs where gradual compression/expansion of spring effectively absorbs mechanical stress (Supplementary Fig. S1), we fabricated scalable PRISWs as strain-engineered soft platforms (Fig. 1a and Supplementary Fig. S2). Compared to the studies on developing various types of island materials (polyimide^14^,^23^,^25^, PET^15^, SU-8^26^, heterogeneous synthetic composite^27^, PDMS^28^,^29^,^30^,^31^, PCB^32^), we adopted poly (methyl methacrylate) (PMMA) as an island material considering robustness and printing-processability. Also, an embedded island structure was addressed to provide flatness of the system for one-step printing of interconnection networks. Specifically, inkjet-printed PMMA islands were enclosed with poly (dimethyl siloxane) (PDMS) matrix in PRISWs. The total thickness of the PRISW was ~50 μm, comparable to that of the human epidermis (50 ~ 100 μm)^33^, and its stretchability allowed wrap-like conformability (Fig. 1a(i,iii)). Owing to the transparency, PRIs were rarely perceived unless illuminated by the tilted light incidence (Fig. 1a(ii)). In virtue of DOD property of inkjet-printing method, various types of PRIs with different size (80 μm, 500 μm, 1450 μm) (Fig. 1b–d) and structure (1- to 5-layered) (Fig. 1b) could be fabricated just in a single batch of the PRISW, simultaneously forming customizable arrangements perfectly matched to the predesigned circuit and functional specification: “dolphin”, “maple leaf”, and letters indicating “ISLAND” and “SNU” were demonstrated in this case (Fig. 1a and Supplementary Fig. S2b). Furthermore, the PRISW could easily be scaled up to a very large area over ~14 inch containing ~3200 PRIs (only limited by the lab equipment specification) and divided into subsections for selective demonstrations, showing superb scalability, cost efficiency and multi-purpose utility (Supplementary Fig. S2c). The embedded PRIs with programmed arrangements successfully served as strain-free building blocks for protecting contact areas of electronic components (Fig. 1d). It is noted that if LEDs or other SMDs are directly integrated onto the soft substrate without embedded islands, a system stress would be entirely applied to the contact areas of electronic components; resulting in electrical and mechanical failure. These PRIs also played a role of spatial backbones for adaptable circuit design similar with a functional node in micro-computer interconnection networks^34^. Based on the optimized PRISWs and *in situ* electronic functionalization, stretchable, skin-conformable electronic circuits with customized designs were implemented (Fig. 1e,f and Supplementary Fig. S3).

### Stability analysis and structural optimization of PRISWs

**Figure 2:**
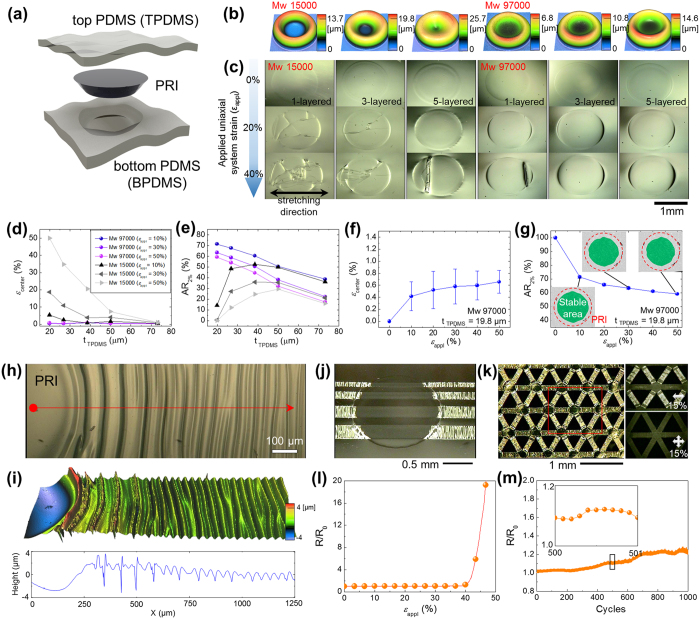
Structural optimization and gradual strain-absorbing design of inkjet-printed stretchable interconnects in PRISW system. (**a**) Schematic illustration of the unit structure of PRISWs comprised of three parts: top PDMS (TPDMS), PRI, and bottom PDMS (BPDMS). (**b**) 3D surface profiler images of 1-, 3-, 5-layered PRIs with Mw 15,000 and 97,000. (**c**) Optical images of elongated PRISWs (applied uniaxial system strain, *ε*_appl_ = 0 ~ 50%) containing 1-, 3-, 5-layered PRIs with Mw 15,000 and 97,000. (**d** and **e**) Effect of top PDMS thickness (t_TPDMS_) and PMMA Mw on the stability of PRISW containing 5-layered PRIs in terms of PRI center strain (*ε*_center_) (**d**) and 2% area ratio (AR_2%_) (**e**). (**f** and **g**) Stability parameters as a function of *ε*_appl_ for optimized PRISW: *ε*_center_ (**f**) and AR_2%_ (**g**). (**h**) Optical image of gradually wrinkled PRISW driven by prestrain (~30%) and UVO_3_ treatment, showing self-constructed gradual strain-absorbing design with a stress-matched amplitude. (**i**) 3D profiler image and corresponding line profile of the wrinkled area described in (**h**). (**j**) Optical image of high-density inkjet-printed Ag interconnects directly fabricated upon the PRISW. Flatness of the PRISW allows direct printing of stretchable conductors, and prestrain-induced gradual strain-absorbing design makes fragile Ag films stretchable. (**k**) Inkjet-printed 2D PRI interconnection networking. (**l**) Electrical characteristics of inkjet-printed Ag interconnect with 50% prestrain as a function of *ε*_appl_ (0 ~ 50%). (**m**) Electrical characteristics of the interconnect under 30% cyclic stretching condition. Stretching speed for (**l**) and (**m**) was equally 10 mm min^−1^.

The analysis on mechanical stability and structural optimization of PRISWs are important for improving the system compactness (or device-to-island fill factor) and conformability by reducing the total thickness while keeping a sufficient level of mechanical reliability. Both of qualitative and quantitative analyses are performed based on the unit structure of PRISWs described in Fig. 2a, which is comprised of top PDMS (TPDMS), PRI, and bottom PDMS (BPDMS). First, we investigated the effect of PRI robustness (PMMA molecular weight, Mw) and structure on the stability. Two different kinds of Mw (15,000 and 97,000) were addressed (PMMA with larger Mw was excluded from comparison due to the excessive viscosity for inkjet-printing). The structural parameters of PRIs were engineered by a few steps of repeated printing that uniformly increased the thickness of crater-shaped PRIs meanwhile keeping the outer diameter fixed (~1.45 mm) through the contact line pinning effect (Figs 1b and 2b and Supplementary Fig. S5); thus, we designated the structure of PRIs as “*N*-step printed PRI” or shortly “*N*-layered PRI” instead of the exact values of structural parameters (Fig. 2b and Supplementary Fig. S6). As shown in Fig. 2c, it is observed that PRIs with Mw 15,000 clearly exhibited fragile characteristics both in single- and multi-layered structures even under small applied uniaxial system strain (*ε*_appl_). By contrast, in a qualitative perspective, the 1-layered PRI with Mw 97,000 possessed the robustness comparable to the 5-layered PRI with Mw 15,000, and the 5-layered PRIs with Mw 97,000 met the optimized stability: keeping their morphologies without deformation even under 50% *ε*_appl_ (Supplementary Fig. S7).

Additional quantitative studies were made to investigate the effect of TPDMS thickness (t_TPDMS_) in conjunction with the comparison between 5-layered PRIs with different Mw. Two kinds of indices were defined to convert the PRI stability to numerical values: (1) PRI center strain (*ε*_center_, maximum principal strain measured at the surface of TPDMS upon the center of PRIs) and (2) stable area ratio in PRI (AR_2%_) that represents the area ratio of specific domain with internal strain <2% to the whole PRI area (Supplementary Fig. S8). The specific value of 2% was adopted as the cracking strain of inkjet-printed silver (Ag) lines (Supplementary Fig. S8a). In case of PRIs (Mw 97,000), it is identified that the decrease in t_TPDMS_ caused drastic increase in AR_2%_ while keeping the stable value of *ε*_center_ (<1%) at the entire *ε*_appl_ regime (0 ~ 50%) (Fig. 2d,e). PRISWs with t_TPDMS_ < 20 μm were excluded from analysis because PRIs were protruded from the PRISW as being strained in most samples. On the other hand, instability in PRIs (Mw 15,000) prevailed in most conditions, showing much higher *ε*_center_ and degraded AR_2%_ (Fig. 2d,e) in all t_TPDMS_ consistent with the fragile characteristics exhibited in Fig. 2c. It is apparent from these results that the PRISW containing 5-layered PRIs (Mw 97,000) with t_TPDMS_ ~20 μm exhibited the optimized mechanical characteristics over the entire *ε*_appl_ regime (0 ~ 50%) for practical wearable applications: *ε*_center_ < 1% (Fig. 2f) and AR_2%_ > 59% (Fig. 2g). To be specific, the optimized PRI was strain-free under the external tensile strain up to 50%, and its maximum strain remains to be smaller than 2% in ~60% area of PRIs.

### Gradual strain-absorbing design of inkjet-printed Ag interconnects

A high-performance stretchable conductor is an essential component for stable operation of electronic devices under skin-like deformations. Numerous efforts have been made to develop advanced materials and/or well-defined strain-absorbing architectures for stretchable conductors: representative approaches include elastic conductors^14^,^35^,^36^,^37^, patterned nanomaterials^38^,^39^,^40^, and metallic conductors with vertical wavy^41^,^42^, serpentine^43^,^44^, and nano-/micro-structure^45^,^46^. Among them, addressing interconnection density, conductivity, robustness, and printing-processability, we adopted a prestrain-induced vertically wrinkled strain-absorbing design of inkjet-printed Ag electrodes (Supplementary Table S2). To figure out the strain-absorbing mechanism of our PRISW, we first formed prestrain-induced wrinkles (SiO_x_/PDMS bilayer structure) on the PRISW in combination with ultraviolet-ozone (UVO_3_) treatment^47^ (Fig. 2h and i): because the wrinkles formed by the UVO_3_-induced SiO_x_/PDMS bilayer structure generally show relatively larger amplitude and wavelength than those formed by the printed Ag/PDMS bilayer structure, strain-absorbing morphology of the PRISW system can be more clearly observed. Interestingly, anisotropic stress distribution in the PRISW system induced by external prestrain (~30%) generated gradual wrinkles along the elongated direction (red arrow in Fig. 2h). In particular, well matched with the theoretical background^48^, localized stress near the PRI boundary caused bifurcated, dual-period wrinkles compared to the single period wrinkle at the far-field region. By contrast, wrinkle formation was suppressed in the PRI area; implying that the PRI accurately served as a strain-free area. From this gradual wrinkling phenomenon or gradual strain-absorbing design with a stress-matched amplitude, it is expected that the stress-localizing instability occurred in rigid-to-soft transition area^49^,^50^ could be overcome with an engineered wrinkle profile compared to the profile with uniform amplitude and wavelength (Fig. 2i).

Addressing the underlying mechanism of gradual strain-absorbing design of UVO_3_-treated PRISW, we fabricated stretchable high-density (minimum width ~50 μm with 10 pL nozzle, ~30 μm with 1 pL nozzle) Ag interconnects simply by one-step inkjet-printing process (Fig. 2j and Supplementary Fig. S9). Similar to the UVO_3_-treated PRISW, inkjet-printed Ag interconnects started to be wrinkled into the gradual strain-absorbing design with the stress-matched amplitude as the prestrain released, whereas the wrinkle formation was highly suppressed in the PRI area. This facile one-step printing and prestrain-induced wrinkling approach easily enabled two-dimensional (2D) stretchable multi-PRI interconnection networking in a triangular lattice configuration (Fig. 2k) while keeping the mechanical and electrical performance (Fig. 2l) and cyclic reliability (Fig. 2m).

### Printing-based automated electronic functionalization directly on prestrained PRISWs

**Figure 3:**
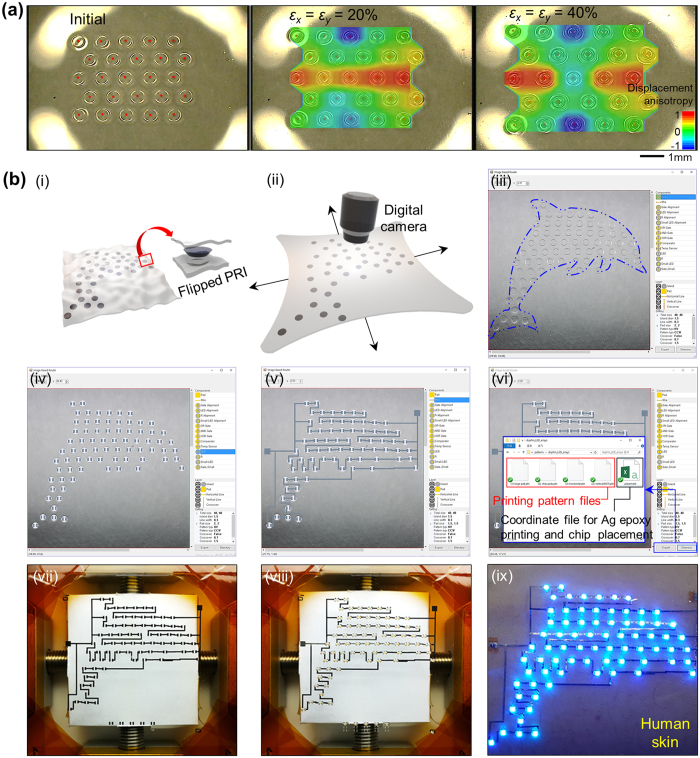
Automated electronic functionalization process on a PRISW. (**a**) Optical images and calculated displacement anisotropy of rearranged PRIs with a sequential biaxial stretching (*ε*_*x*_ = *ε*_*y*_ = 0 ~ 40%). Displacement anisotropy is defined as “(*x* displacement − *y* displacement)/(*x* displacement + *y* displacement)” (**b**) Experimental details on automated electronic functionalization process from capturing image to fabricating customized electronic wrap: (i) An appropriate PRISW is prepared. (ii) The PRISW is biaxially prestrained (*ε*_*x*_ = *ε*_*y*_ = 25–30%), and then the expanded state of the sample is captured by a digital camera. (iii) The captured image is loaded to the home-made circuit networking program as a background image. (iv) The modified positions of each PRI are automatically pinpointed with functional elements (LED in this case). (v) Customized circuit was designed and drawn by bridging the pinpointed PRIs. (vi) The given coordinates of the interconnection networks and functional chips were converted to the printing pattern files and coordinate files for Ag epoxy printing and chip placement via our program. (vii) The interconnection network designed in the previous step was fabricated directly on the PRISW via an inkjet-printing process. (viii) Functional IC chips and other surface-mountable device (SMD) components were automatically placed by using a chip placement machine in conjunction with robust Ag epoxy bonding. (ix) Finally, prestrain was released to form spontaneous 2D wrinkles in the overall system.

In spite of fruitful advantages of the prestrain-induced strain-absorbing design concept, the prestrain generally provokes anisotropic displacement of spatially distributed rigid islands (Fig. 3a) and also causes an alignment issue on implementing desired electronic systems upon PRISWs; thereby hindering a use of predefined masks and associated common methodologies. Indeed, prestrain-induced anisotropic rearrangements of PRIs are exacerbated as the number and density of PRIs increase. In large-area applications, therefore, prestrain-based direct fabrication approach has thoroughly been avoided. Here, based on the PRISW concept, we introduce a novel method that clearly solves the issue on anisotropic displacement of PRIs without losing both scalability and printing-based *in situ* processability. The key idea is to use the captured image of the prestrained PRISW as the background image for circuit design process (Fig. 3b(i–iii)). For our demonstrations, biaxial prestrain level was set as 25~30% based on the elastic property of the skin itself, which typically shows an elastic response to tensile strain <30% and irreversible effects under strains beyond 30%^51^. Based on the home-made automated circuit networking program, spatially rearranged PRIs were automatically pinpointed as spatial backbones by image processing (Fig. 3b(iv)), and then customized circuit layout were drawn by bridging the PRIs in an arbitrary manner (Fig. 3b(v)). Since the whole circuit was configured at a prestrained state based on the exact image, a misalignment issue could be avoided. Moreover, the exact coordinate data of interconnection networks and electronic components evaluated from the rearranged PRI positions were automatically extracted (Fig. 3b(vi)) to facilitate sequentially followed steps of printing Ag interconnects (Fig. 3b(vii)), printing Ag epoxy for strong adhesion between printed pads and to-be-placed electronic units, and integrating functional SMD chips (Fig. 3b(viii) and Supplementary Fig. S10); successfully converting a PRISW into the functional electronic wrap (dolphin display in this case). Experimental details appear in Methods. This novel, automated electronic functionalization process has significance in (1) automated and sequentially connected process steps, (2) printing-based rapid, scalable, low cost process, (3) multi-functionality with versatile SMD chip integration, and (4) unlimited design freedom in circuit design.

### Rapid implementation of customizable electronics on skin

**Figure 4:**
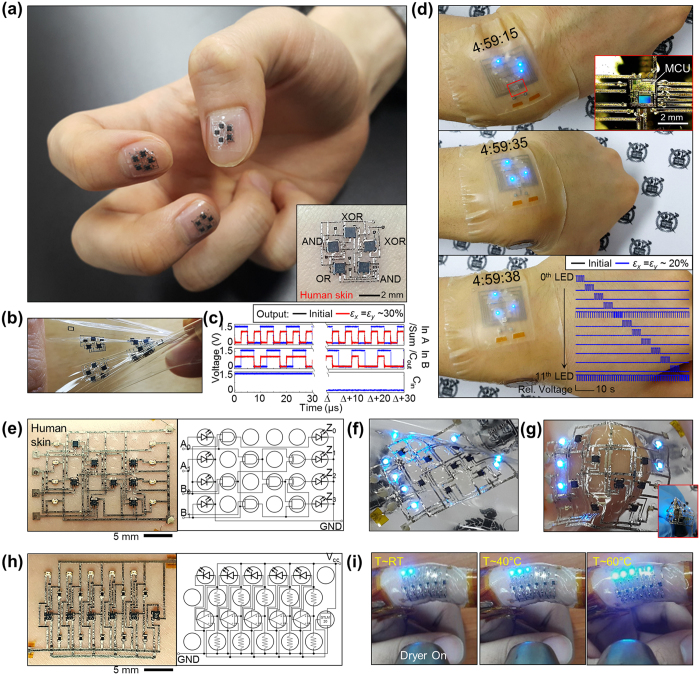
Rapid implementation of versatile customizable electronics on skin. (**a**) Photograph of digital full adders with a customized design directly fabricated on the PRISW which contains pentagonal PRI arrangements. Owing to the wrap-like conformability, the full adders are easily adhered to human nails and skin (inset image). (**b**) Photograph of stretched/twisted full adders. Several full adders can be fabricated in a single batch. (**c**) Input/output characteristics of full adders both at an initial and stretched state (*ε*_*x*_ = *ε*_*y*_ = ~30%) (blue line: input signals, black and red line: output signals). (**d**) Photographs of the wearable analog watch operation under sequential wrist movements. Top inset: Enlarged optical image of the wire-bonded MCU region. Bottom inset: Operating input signals of twelve LEDs before and after deformation under 20% biaxial strain. (**e**) Optical image and corresponding logic diagram of 2-bit multiplier laminated on the human skin. (**f** and **g**) Photographs of the stretched 2-bit multiplier indicating “3 × 3 = 9” (**f**), and wrapped around a finger (“3 × 1 = 3”) and a ball pen (inset image) (**g**). (**h**) Optical image and corresponding logic diagram of temperature monitoring system. (**i**) Photographs of sequential operation of the temperature monitoring system as a dryer approached (temperature was increased from room temperature (RT) to around 60 °C).

Based on the two key aspects of PRI-based soft substrate engineering and printing-based automated electronic functionalization, we demonstrate multiple types of functional electronic wraps that involve customizable circuit layouts to provide evidence for the potential widespread uses of our work. One of the strongest assets of our strategy is the feasibility of rapid implementation of high-density skin-conformable electronic systems within a self-computable and fully-integrated form (that is, no external wires, external operating circuits, nor external processors for computation and analysis are needed). A high-density interconnection network with a gradual strain-absorbing design increases system compactness. In addition, the capability of direct integration of multiple types of IC chips and electronic units allows various functions and self-computation without external wirings for additional computation. Although large size IC chips can be integrated into PRISWs (Supplementary Fig. S11), system functions are intended to be segmented into a variety of small components (<1 mm) to minimize bulky characteristics induced by rigid chips and to improve skin-conformability. As a fundamental unit of digital computer, we demonstrate electronic wraps that function as a digital full adder in a highly compact design based on the pentagonal PRI arrangement, dimension of which is much smaller than human nails (Fig. 4a). Owing to the gradual strain-absorbing design of interconnection networks and reduced thickness of the optimized PRISW, the full adders show good skin conformability (inset image of Fig. 4a), deformability (Fig. 4b) and stretchability (~30% biaxial strain) (Fig. 4c), which were attributed to the stress-shielding effect of PRIs and the wrinkled geometry of printed Ag interconnects (Supplementary Fig. S12). From a technical viewpoint, printable crossovers were applied to facilitate partially-multilayered circuit designs and to improve the system compactness (Supplementary Fig. S12a).

As practical applications, wearable display including analog watch and passive matrix (PM) display were also demonstrated in a similar manner based on the appropriate PRI arrangements (Fig. 4d and Supplementary Figs S13 and S14). Specifically, the watch and PM display were comprised of a micro-controller unit (MCU) programmed for adequate operation, numbers of LEDs (12 for watch and 35 for PM display), and a thin 3 V battery. All electronic components were protected by PRIs and functionally bridged by inkjet-printed stretchable interconnects with customized layouts. While all LEDs were tightly bonded with inkjet-printed Ag pads via Ag epoxy, the MCU chip was directly connected to Ag pads by means of wire-bonding technology owing to the tiny size of MCU chip pads (top inset image in Fig. 4d). Both of wearable customized display systems were stably operated under harsh conditions like sequential wrist movements and also exhibited good conformability to the skin (Supplementary Fig. S14).

Feasibility of user-controllable selection of PRIs for desired functions and layouts further supports the universality of our strategy. Inspired by conventional universal PCB, we introduced a concept of universal PRISW that contains lattice-like PRI array with adequate numbers and inter-PRI spacing (Supplementary Fig. S15a); for the first time realizing ultimate customization of skin-conformable electronics from substrate technology (PRI arrangement), circuit design (PRI selection and interconnection networks) to functional implementation (versatile chip integration). Based on this universal PRISW concept, we demonstrated fully printed, medium-scale (~22 × 16 mm^2^ with 16–21 electronic components) ultimately customizable electronic wraps, whose specific functions could be diversified in a customizable manner; here, a 2-bit digital multiplier (Fig. 4e–g) and temperature monitoring system (Fig. 4h,i) were demonstrated on skin. It is noted that the number of PRIs and thus the size of universal PRISW can be easily scaled up (Supplementary Fig. S2c), implying that larger-scale application-specific electronic wraps can be demonstrated in the same manner. The skin-conformable 2-bit digital multiplier was comprised of 8 logic gates and 8 LEDs connected by inkjet-printed wrinkled Ag interconnects and 26 crossovers (Fig. 4e and Supplementary Fig. S15b–d). The multiplier also showed stable operation under harsh conditions: stretched (~20%) (Fig. 4f), wrapped around the human finger and stabbed by the ball pen (radius < 1 mm) (Fig. 4g). The skin-conformable temperature monitoring system was comprised of temperature sensor IC, 5 comparators, 10 resistors for voltage dividing, and 5 LEDs for output display (Fig. 4h and Supplementary Fig. S15e). Each LED was sequentially turned on at 5, 18, 31, 44, 56 °C by exact comparison between the reference voltage and output voltage of the temperature sensor that linearly decrease as temperature increases (Supplementary Fig. S15f,g). As shown in Fig. 4I, temperature monitoring LEDs worn onto the finger were sequentially turned on as a hair dryer approached, indicating that surrounding temperature was varied from room temperature (~25 °C) to around 60 °C.

## Discussion

In conclusion, the term “customization” is introduced and realized in the field of stretchable electronics as one of the essential feature for bridging the gap between application-specific research and adaptable, multidisciplinary uses of such state-of-the-art electronic systems. Based on the concept of strain-engineered electronic wrap, we developed a rapid, universal methodology for implementing customizable electronic on skin. Specifically, the methodologies to form multilayered PRI structure and corresponding PRISW with optimized stability and to implement printing-based electronic functionalization at a prestrained state were developed. The mainly-utilized inkjet-printed Ag interconnects with a gradual strain-absorbing design fulfills a sufficient level of conductivity (~9 × 10^4^ S/cm), stretchability (~50%), cyclic property (>1000), and interconnection density. Furthermore, the newly developed automated circuit design process resolved all major impediments and greatly reduced process time (a couple of hours taken for system implementation). Versatile applications based on the strain-engineered electronic wrap concept supported the universality of our strategy. It is expected that this work would establish a fundamental framework for broad, adaptable uses of stretchable electronics.

## Methods

### Specification of surface-mountable device (SMD) chips

All packaged chips utilized in this work are SMD-type and commercially available. Dimensions, part numbers and manufacturers of SMD chips are followed: LED (1 mm × 0.6 mm × 0.2 mm, SML-P11-UT, Rohm Semiconductor), AND gate (1 mm × 1 mm × 0.4 mm, 74AUP1G08, NXP Semiconductors), OR gate (1 mm × 1 mm × 0.4 mm, 74AUP1G32, NXP Semiconductors), XOR gate (1 mm × 1 mm × 0.4 mm, 74AUP1G86, NXP Semiconductors), resistor (1 mm × 0.5 mm × 0.4 mm, MCR01, Rohm Semiconductor), comparator (1 mm × 1 mm × 0.5 mm, NCX220GS, NXP Semiconductors), temperature sensor IC (1.3 mm × 1 mm × 0.5 mm, STLM20, STMicroelectronics), MCU dye (2 mm × 2 mm × 0.4 mm, PIC16LF1509, Microchip Tech.).

### Fabrication of printed rigid island embedded soft wrap (PRISW)

5 wt% poly (vinyl alcohol) (PVA) (Sigma Aldrich, average Mw 31000-50000, 87–89% hydrolyzed) solution which can be dissolved in deionized (DI) water was spin-coated upon the cleaned glass substrates (700 μm thickness), and the coated layer was thermally annealed for 1 hr at 90 °C. Subsequently, poly (dimethylsiloxane) (PDMS) elastomer (Sylgard 184, Dow Corning), mixed at 20:1 ratio, was poured onto the PVA-coated glass substrate, and spin-coated for the top PDMS layer. The coated substrate was then thermally cured for 2 hr on a 125 °C hot plate. The thickness of the PDMS membrane was ~20 μm. For PRI fabrication, we used 4 wt% poly (methyl methacrylate) (PMMA) (Sigma Aldrich, Mw ~97000) solution dissolved in propylene glycol monomethyl ether acetate (PGMEA) (Sigma Aldrich, ≥99.5%). To improve the adhesion between the substrate and the ink, ultraviolet-ozone (UVO_3_) treatment was carried out (power = 29 mW cm^−2^) for 5 min before printing. PMMA solution printing was successfully implemented onto the surface-treated PDMS membrane by using a piezoelectric inkjet-printer (DMP-2831, Dimatix Corp.) with 6 nozzles whose size is 21 μm. To be specific, stepped voltage was applied to construct a fine jetting profile, and the jetting velocity was ~8 m/s. To fabricate well-defined PRIs, we adopted multistep printing technique. Owing to its maskless DOD property, the size and position of PRIs were easily tunable. As-printed PMMA PRIs were heated on a 90 °C hotplate for 1 hr to evaporate residual solvents. After an additional 5 min of UVO_3_ treatment for enhancing the adhesion between PDMS and PMMA PRIs, the upper PDMS was spin-coated as the bottom PDMS layer. Finally, the sample was immersed in DI water to dissolve the sacrificial layer for freestanding, then flipped to obtain the PRISW. The total thickness of the PRISW was ~50 μm (The whole process flow is depicted in Supplementary Fig. S2a).

### Automated circuit design process

The total procedure was comprised of nine steps, all of which were sequentially connected and available in an *in*-*situ* manner (Fig. 3b): Step 1, the appropriate PRISW was prepared (Fig. 3b(i)). Step 2, the PRISW was two-dimensionally (2D) prestrained (*ε*_*x*_ = *ε*_*y*_ = 25–30%), and then the expanded state of the sample is captured by a digital camera (Fig. 3b(ii)). Step 3, the captured image was loaded to the home-made circuit networking program as a background image (Fig. 3b(iii)). Step 4, the modified positions of each PRI were automatically designated with functional elements (LED in this case) (Fig. 3b(iv)). Step 5, customized circuit was designed and drawn by bridging the designated PRIs. Since the captured image of the prestrained PRISW in the previous step provided pixelated coordinates of the rearranged system, those of interconnection networks and electronic components that are configured upon the captured image by the program can be evaluated (Fig. 3b(v)). Step 6, the given coordinates of the interconnection networks and functional chips were converted to the printing pattern files and coordinate files for Ag epoxy printing and chip placement via our program (Fig. 3b(vi)). Step 7, the interconnection network designed in the previous step was fabricated directly on the PRISW via an inkjet-printing process (Fig. 3b(vii)). By virtue of a facile UVO_3_ treatment (~22 min), the wettability of Ag ink (ANP corp.) was greatly improved; as a result, inkjet-printed large-area Ag interconnects with fine quality could be obtained. Step 8, functional IC chips and other surface-mountable device (SMD) components were automatically placed by using a chip placement machine (TM220A, NeoDen Tech) in conjunction with robust Ag epoxy bonding (Fig. 3b(viii)). Step 9, prestrain was released to form spontaneous 2D wrinkles in the overall system (Fig. 3b(ix)). After 2D wrinkles were completely formed, a liquid PDMS (30:1 ratio) was poured and spin-coated for reliable device packaging (Supplementary Fig. S16).

### Analysis on surface strain profile of PRISWs using DIC

Upon the as-prepared PRISW, easily accessible toner particles or baking powders (average particle size ~20 μm) were dredged as speckle patterns. Also, dark background was prepared to enhance the contrast level. Then, the speckle-patterned image was sequentially captured by optical microscope as the PRISW was slowly elongated. Each image had a resolution of 720 × 480 pixels. The sequential image frames were analyzed by comparing the relative movement of each speckle pattern, easily available by an image-processing tool (Vic-2D 2009, Correlated Solutions); then, displacement field data were automatically converted into Hencky strain values for maximum principal strain (Supplementary Fig. S4).

### Robust SMD chip bonding with Ag epoxy

The total procedure for SMD chip bonding was comprised of four steps, all of which were available in an *in situ* manner (Supplementary Fig. S10). In step 1, a proper layout of inkjet-printed Ag pad that was designed relatively larger than SMD contact pads was *in situ* fabricated inside the PRI region at a prestrained state. In step 2, two kinds of epoxy (Ag epoxy and pure epoxy) was printed at exact positions by the automatic dispenser (SHOTmini 200Sx, Musashi Engineering, Inc.). Specifically, pure epoxy was printed as a bank to prevent electrical short, and then Ag epoxy was printed directly onto the prepared pad layout for a robust contact. The amount of Ag epoxy was appropriately controlled according to the dimension of SMD contact pads. In step 3, functional chips including LEDs, logic gates, resistors, comparators, and temperature sensors were picked and placed by the chip placement machine at exact positions (displacement error ~25 μm). After that, the sample was annealed on a 160 °C hotplate for 30 min. In the last step, the prestrain was released for generating well-defined wrinkles. Owing to the stress-shielding effect, wrinkles were suppressed inside the PRI. The completely bonded SMD chips exhibited a superb robustness even under an extreme deformation (Supplementary Fig. S10c).

## Additional Information

**How to cite this article:** Byun, J. *et al*. Fully printable, strain-engineered electronic wrap for customizable soft electronics. *Sci. Rep.* **7**, 45328; doi: 10.1038/srep45328 (2017).

**Publisher's note:** Springer Nature remains neutral with regard to jurisdictional claims in published maps and institutional affiliations.

## Supplementary Material

Supplementary Information
