# Supplementary material for: Fully printable, strain-engineered electronic wrap for customizable soft electronics
Source: Sci Rep. 2017 Mar 24;7:45328. doi: 10.1038/srep45328 (PMC5364427; doi:10.1038/srep45328)
Supplement: Supplementary Information [file srep45328-s1.pdf]

Supplementary Information

Fully printable, strain-engineered electronic wrap for customizable soft electronics

Junghwan Byun†, Byeongmoon Lee†, Eunho Oh, Hyunjong Kim, Sangwoo Km, Seunghwan Lee, and Yongtaek Hong*

Department of Electrical and Computer Engineering, Inter-University Semiconductor Research Center (ISRC), Seoul National University, 1 Gwanak-ro, Gwanak-gu, Seoul 151-744, Korea

†These authors contributed equally to this work.

*Corresponding author (e-mail: yongtaek@snu.ac.kr)

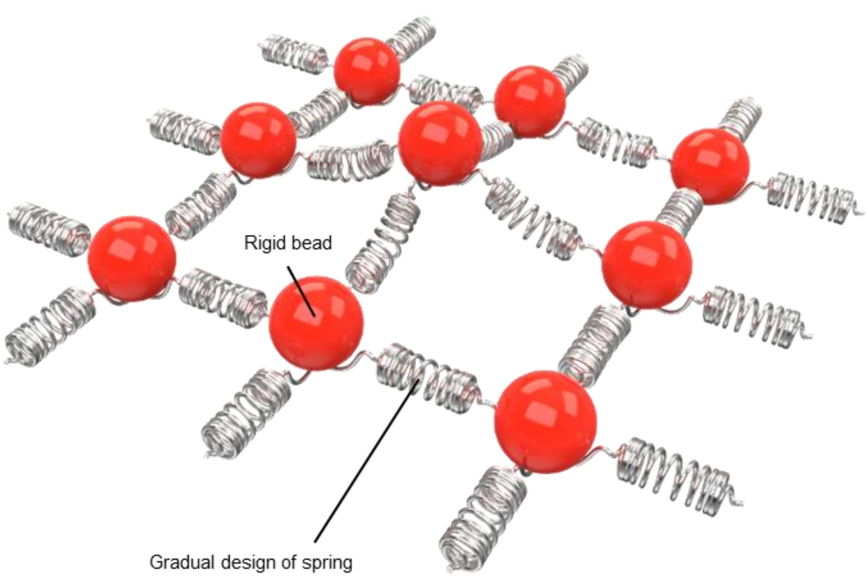

Figure S1. Conceptual schematic of stretchable bead network. The bead and spring represent the PRI and inkjet-printed stretchable interconnect with gradual strain-absorbing design, respectively. Gradual design of the spring network where gradual compression/expansion absorbs mechanical stress is exactly matched with the vertically wrinkled gradual strain-absorbing design of the printed interconnects with a stress-matched amplitude.

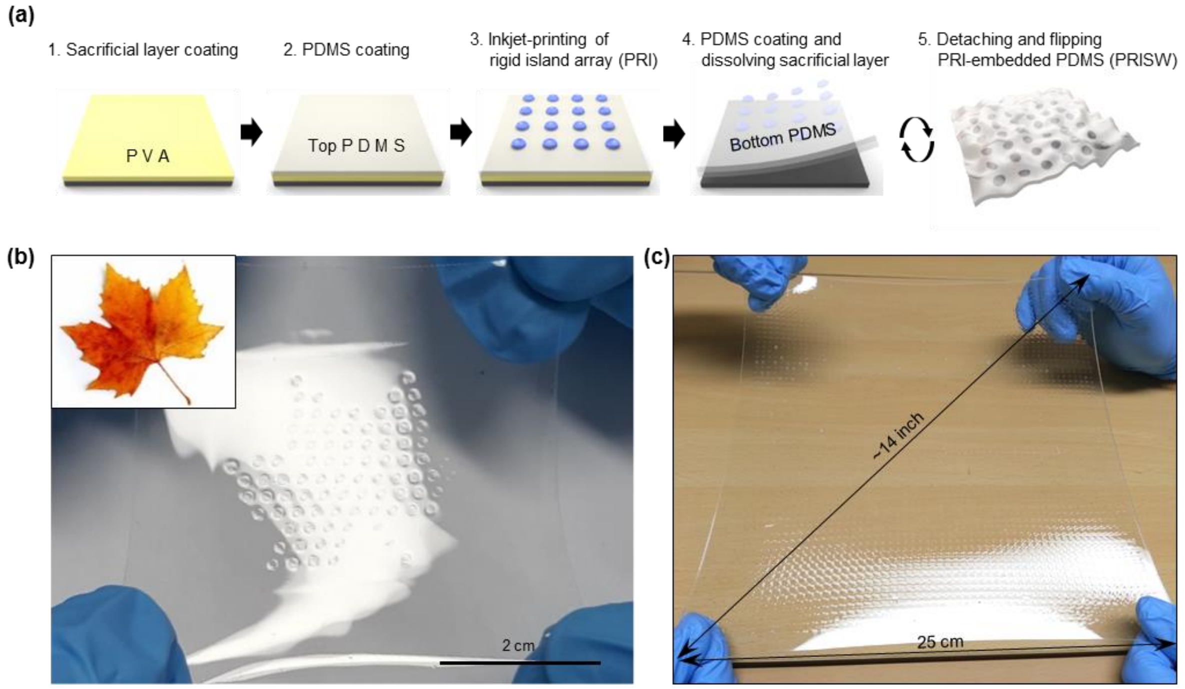

Figure S2. Preparation of large-area, customizable PRISWs. (a) Fabrication process of the PRI-embedded soft wrap (PRISW). (b) Photograph of the PRISW representing a "maple leaf". (c) Photograph of the very large-scale PRISW (~14 inch, ~3200 PRIs). This 14 inch PRISW, size of which was limited only by the lab facility, shows facile scalability of our universal PRISW approach, and it could be divided into several pieces of univesal PRISWs containing a specific amount of PRI arrays.

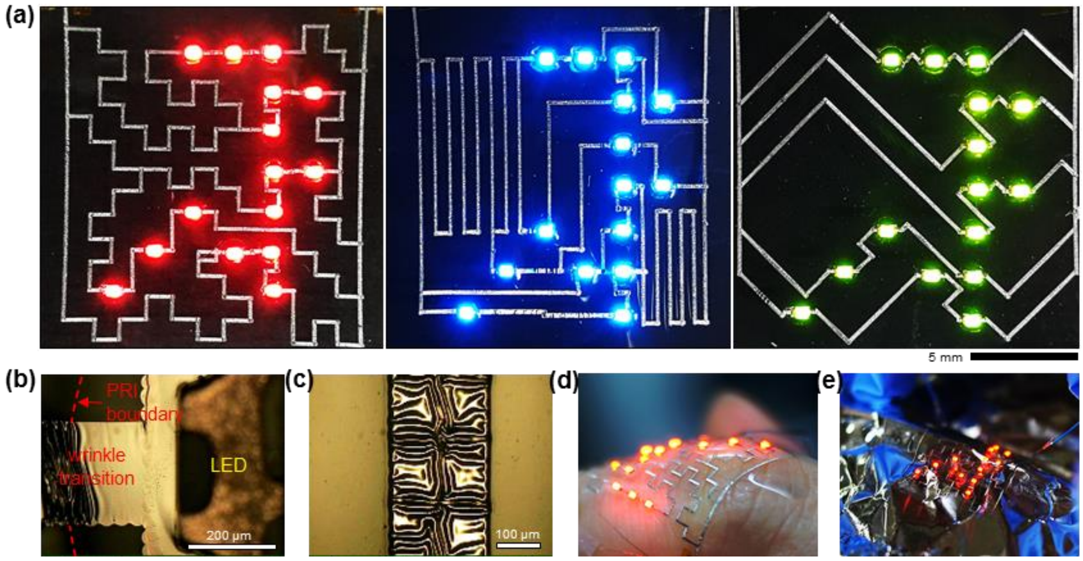

Figure S3. Demonstrations of customizable circuit design with inkjet-printed stretchable interconnection networks. (a) Photographs of three different layouts of stretchable display characterized by different LED colours and customized interconnection networks with different set of LEDs. (b and c) Optical images of the selected areas of the system: the rigid-to-soft transition area with a wrinkle-transition phenomenon (b) and the wrinkled interconnect (c). (d and e) Photographs of the stretchable display laminated onto the human finger (d) and the crumpled Al foil (e).

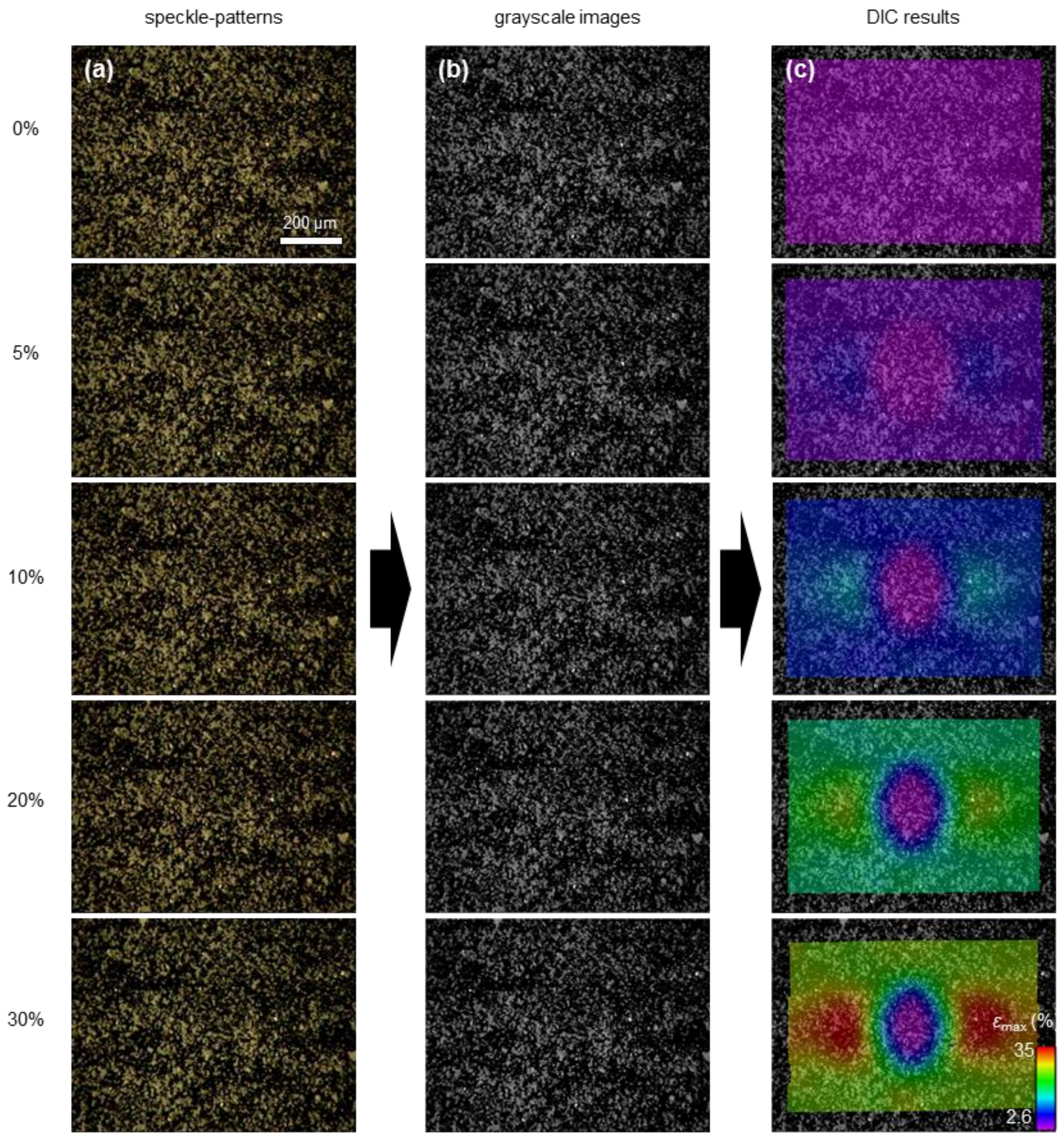

Figure S4. Process flow of digital image correlation (DIC) method. (a) First, randomly distributed speckle patterns (baking powder, average size ~ 20 μm) are uniformly formed, and then the interesting region is captured by an optical microscope or digital camera. (b) Second, the captured images are transformed into grayscale to enhance the contrast level. (c) Finally, the sequential image frames are analyzed by an image-processing tool (Vic-2D 2009, Correlated Solutions) to evaluate the maximum principal strain.

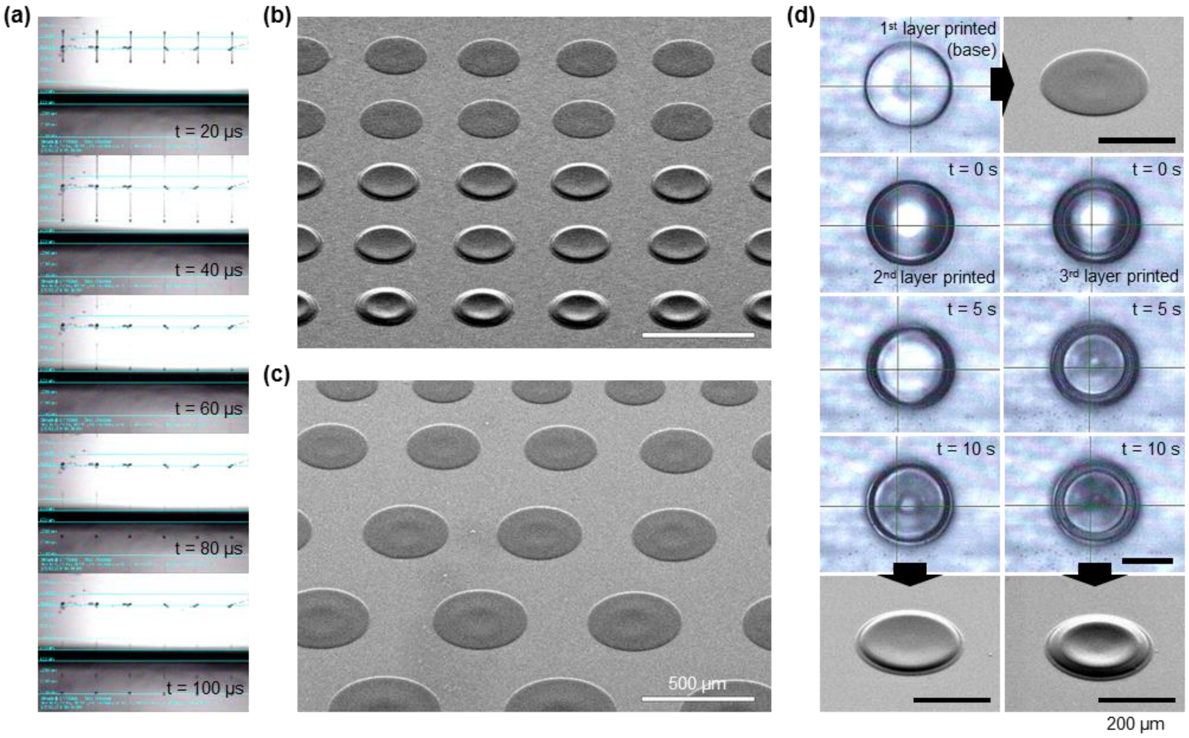

Figure S5. Fabrication of inkjet-printed rigid islands (PRIs). (a) Sequential image frames of PMMA ink jetting. Time interval, 20 μs. (b and c) SEM images of PRI array with different printing steps (1 to 3 steps) (b), and different size (250 to 700 μm) (c). (d) Sequential image frames of real-time formation process of an individual PRI and corresponding SEM images. Time interval, 5 s. Scale bars, 200 μm.

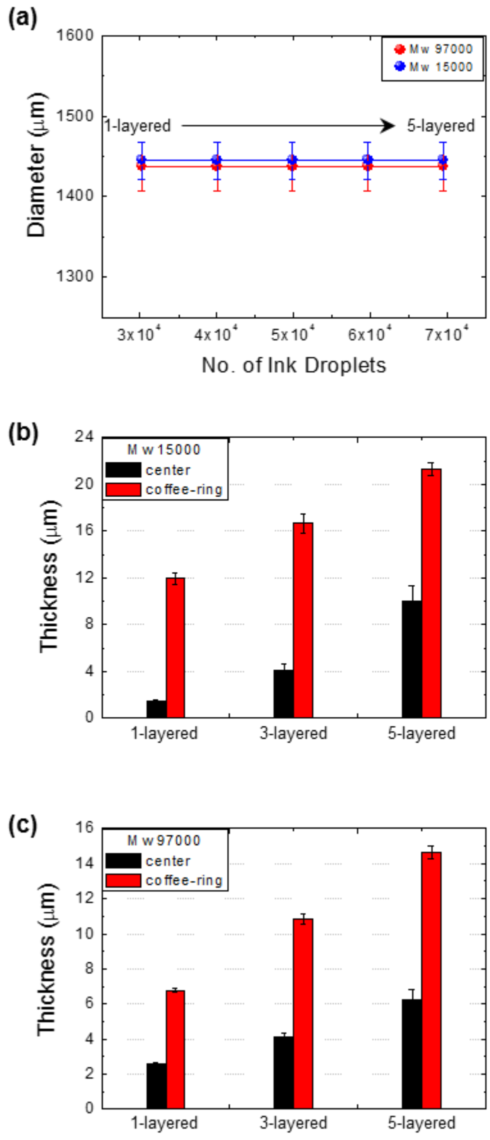

Figure S6. Structural parameters of multistep-printed PRIs. (a) Size dependency of PRIs on printing steps or the amount of ink droplets. (b and c) Center and coffee-ring thickness of multi-layered PRIs of Mw 15,000 (b) and 97,000 (c).

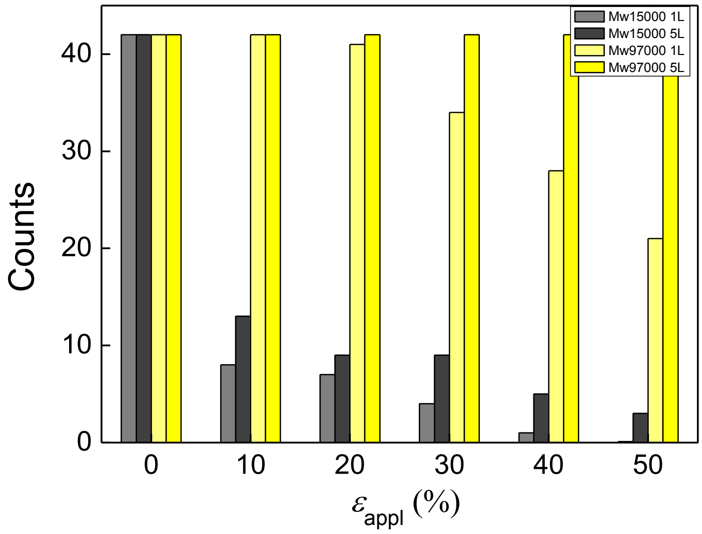

Figure S7. Cracking tendency of PRIs with different structure and Mw. Starting from 42 PRIs for each condition, the number of intact PRIs is counted together with increasing applied uniaxial system strain (ε_{appl} = 0 ~ 50%). Both of molecular chain length and structural parameter affect the stability of the PRI and PRISW.

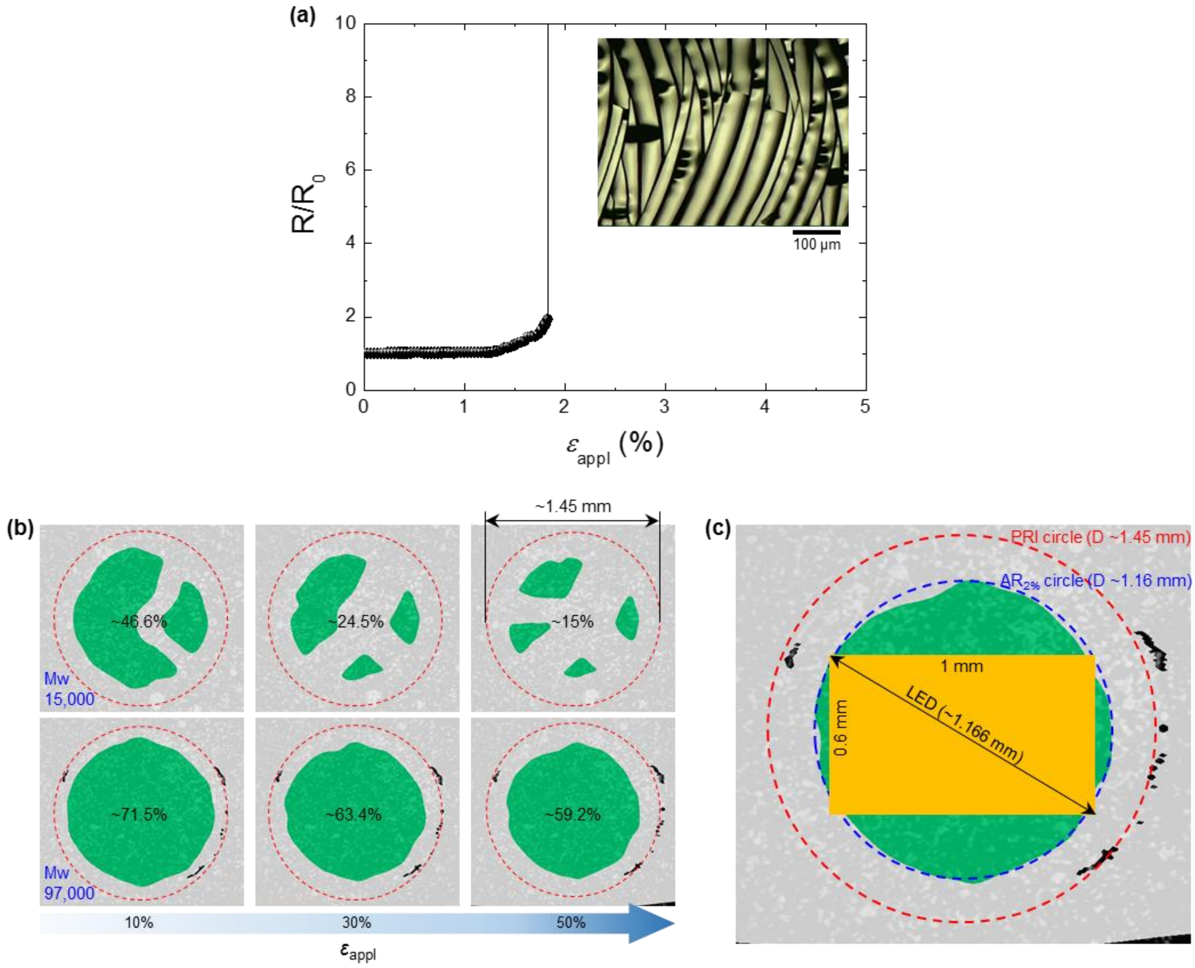

Figure S8. Detailed description on AR$_{2\%}$. (a) Electrical characteristics of inkjet-printed Ag electrode directly fabricated on the bare PDMS. Cracking strain is around 2% (inset image: optical image of the cracked Ag film under the small strain). (b) AR$_{2\%}$ of the PRISW (PRI diameter ~1.45 mm, thickness of TPDMS (t_{TPDMS}) ~20 μm, 5-layered PRI) with different PRI robustness (PMMA Mw 15,000 and 97,000). Surface strain is evaluated from DIC, and AR$_{2\%}$ is calculated by image processing. (c) AR$_{2\%}$ of the PRISW in (b) under 50% ε_{appl} showing a sufficient amount of strain-free area that can cover the SMD chip LED whose dimension is 1 x 0.6 x 0.2 mm^3.

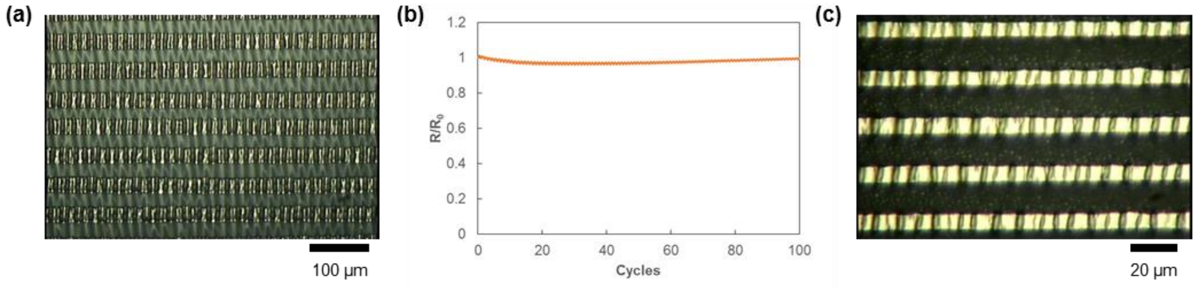

Figure S9. Minimum feature size of inkjet-printed wrinkled interconnects. (a) Optical image of the wrinkled interconnects fabricated by using a 1 pL cartridge of the inkjet-printing system (DMP-2831, Dimatix Corp.). (b) Cyclic reliability of the interconnects in (a). (c) Optical image of the finer interconnects fabricated by using a super inkjet printer (SIJ-S050, SIJTechnology Inc.).

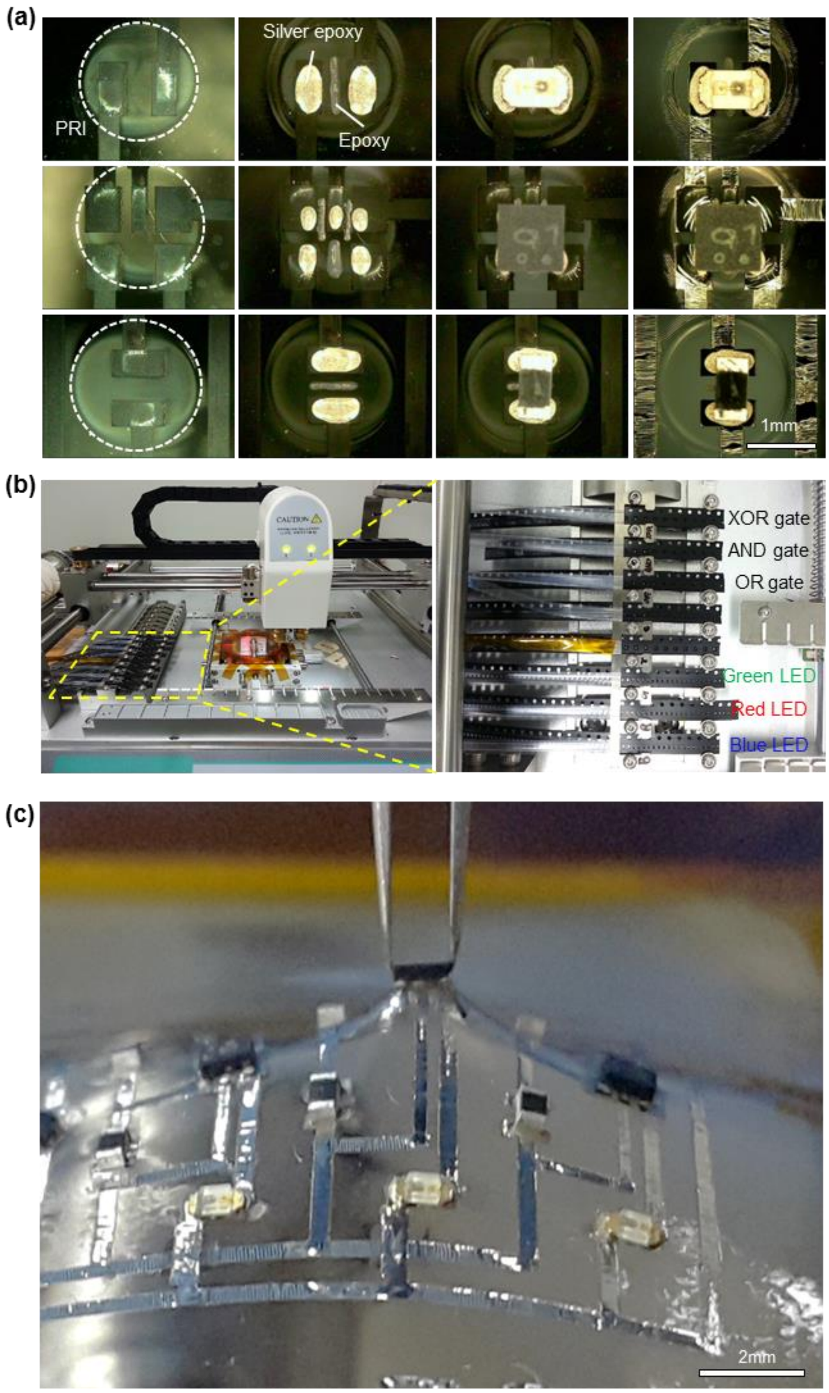

Figure S10. Functional chip bonding via Ag epoxy printing directly on the PRISW. (a) Process flow of Ag epoxy bonding with the SMD LED, logic gate, and resistor: Firstly, a proper layout of Ag pad was prepared inside the PRI region. Next, two kinds of epoxy (Ag epoxy and pure epoxy) were printed and functional SMD chips were accurately placed by a chip placement machine. After that, the sample was annealed and the prestrain was released for generating wrinkles. (b) Photographs of the whole system layout of SMD chip placement

machine and the prepared SMD chips in tape and reel packaging including digital logic gates (XOR, AND, OR) and LEDs with different colours. (c) Photograph showing the robustness of the Ag epoxy bonding.

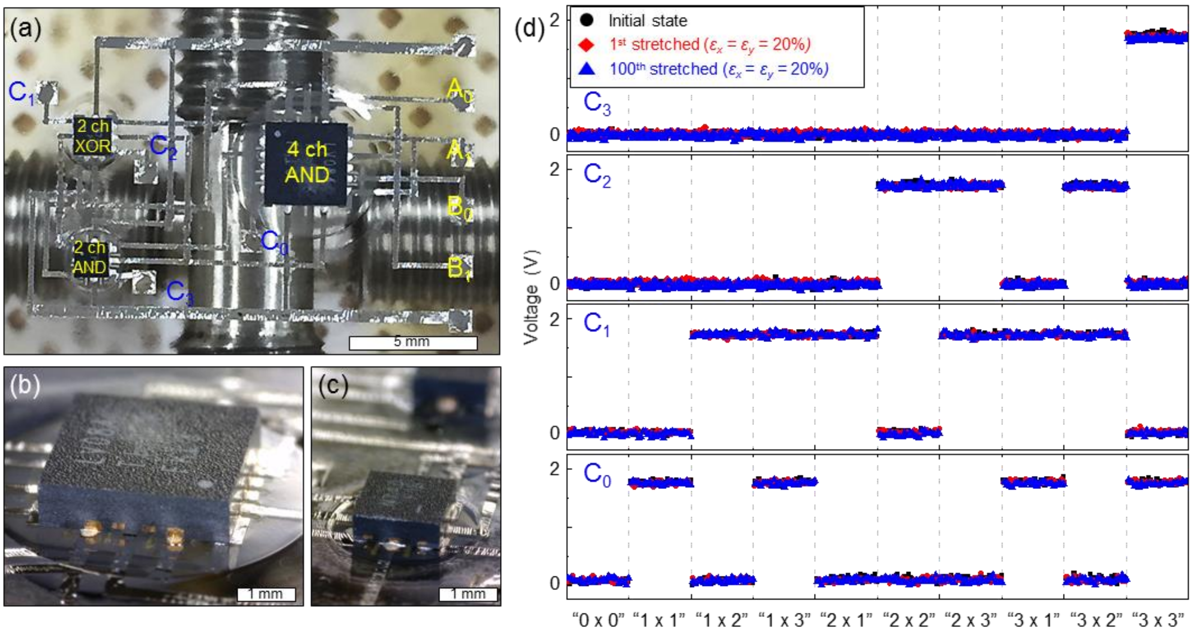

Figure S11. A bulky, stretchable 2-bit multiplier based on a large PRI (diameter ~5.6 mm). (a) Photograph of the whole system comprised of 4-channel AND gate (SN74LV08ARGVR, Texas Instruments (TI), 3.5 x 3.5 mm^2), 2-channel AND gate (SN74AUP2G08, TI, 1.5 x 1.5 mm^2), and 2-channel XOR gate (NC7WZ86, Fairchild Semiconductor, 1.6 x 1.6 mm^2). (b) Magnified optical image of 4-channel AND gate mounted on the large size PRI (diameter ~5.6 mm). (c) Magnified optical image of 2-channel XOR gate mounted on the medium size PRI (diameter ~2.5 mm). (d) Output characteristics (C_0 ~ C_3) of initial, stretched, and 100-times stretched multiplier in multiple states (A x B). The system was equi-biaxially stretched at a speed of 1 mm s^{-1}.

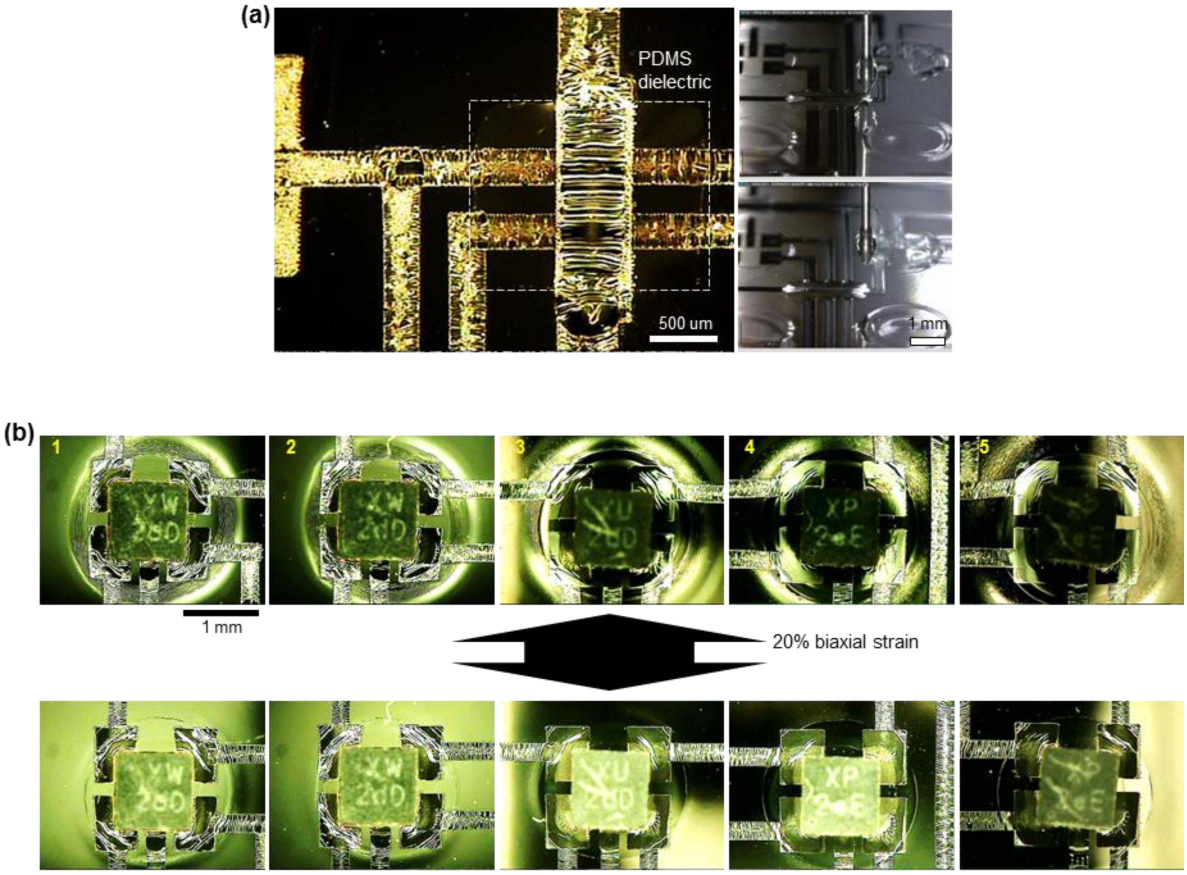

Figure S12. Inkjet-printed crossover and stress-shielding effect of PRIs. (a) Optical image of the inkjet-printed stretchable crossover with a wrinkled geometry (left), and photographs of the home-made dispensing system that consists of a nozzle (inner diameter ~60 μm), dispensing machine and XYZ-moving stage. (b) Optical images of individual logic gates safely protected by PRIs. Wrinkle-transition phenomena near the PRI boundary support the stress-shielding effect of PRIs.

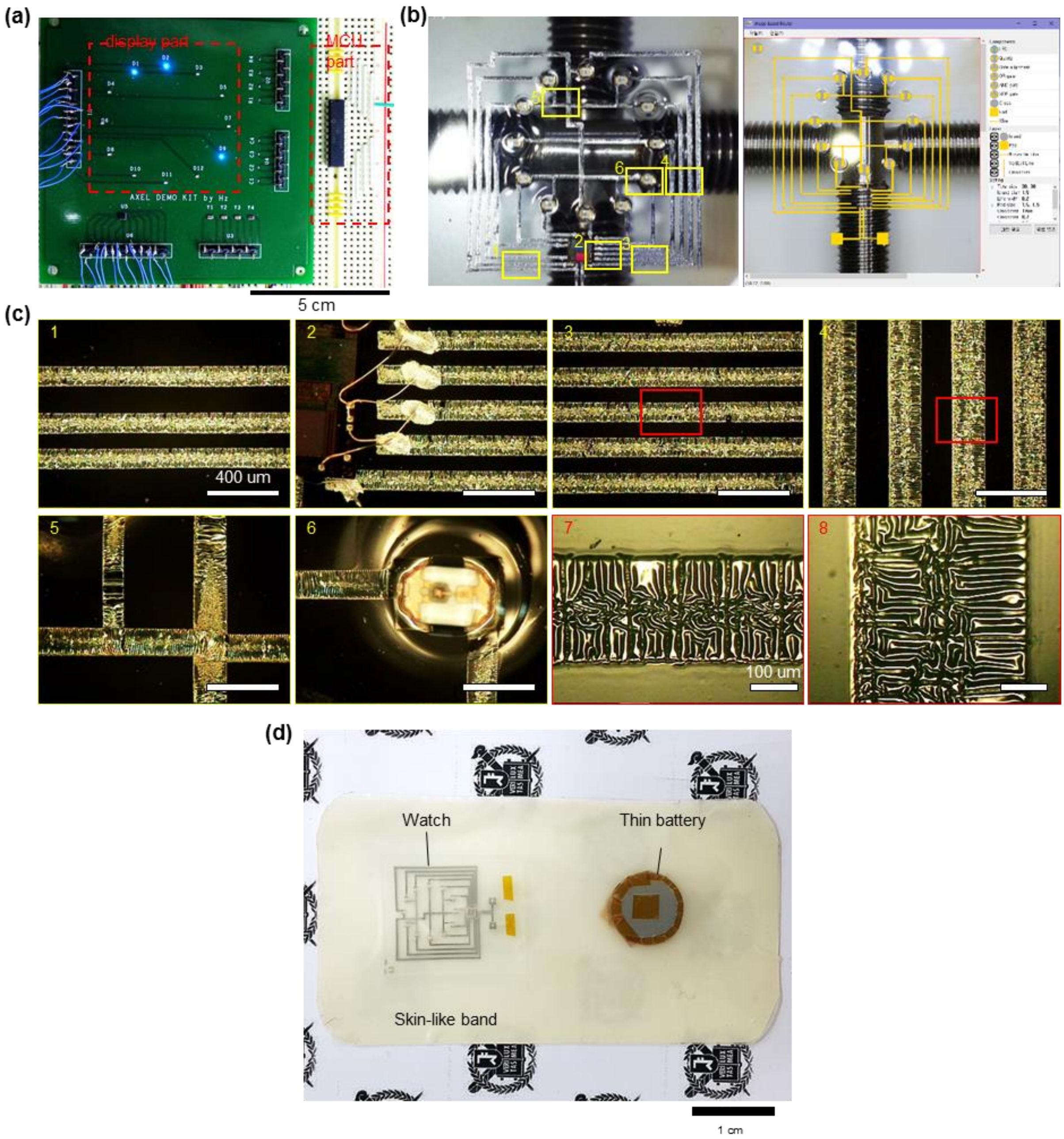

Figure S13. Customized wearable analog watch. (a) Photograph of the watch in a rigid PCB form. (b) Photograph (left) and screenshot image (right) of the customized wearable watch circuit layout after being released (prestrain ~25% biaxial strain). (c) Magnified optical images of the wrinkled Ag interconnects. In overall areas, well-defined wrinkles are generated on printed Ag interconnects. (d) Photograph of the all-in-one skin watch system.

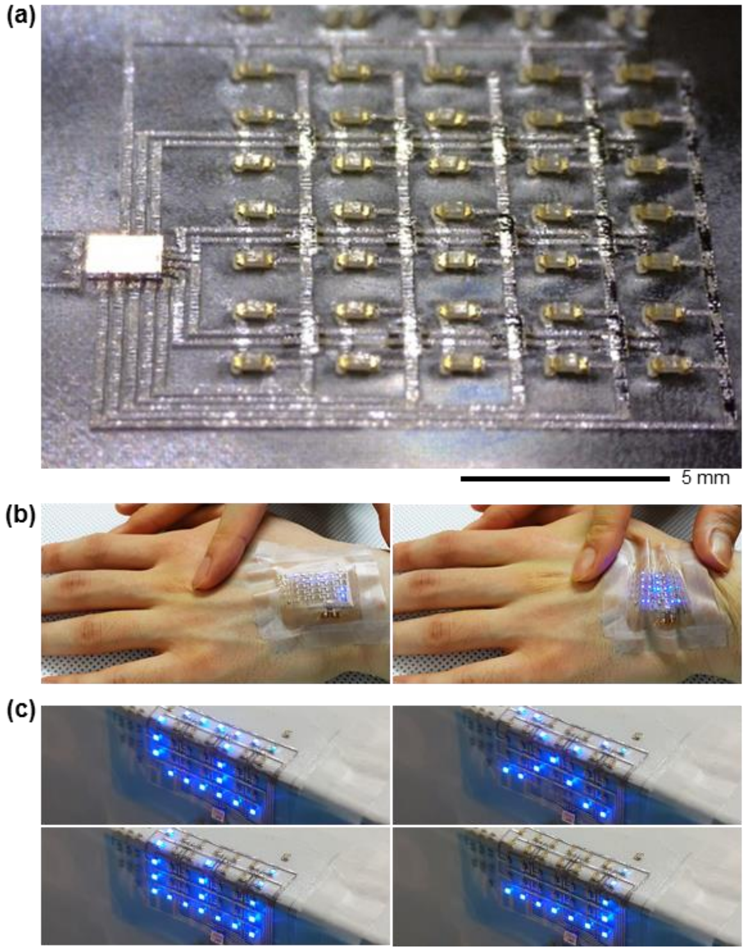

Figure S14. Customized wearable passive matrix (PM) display. (a) Photograph of the whole PM display circuit consisting of a MCU and 35 LEDs. (b and c) Photographs of the PM display deformed like human skin (b) and folded (c) while exhibiting sequential letters “AXEL”.

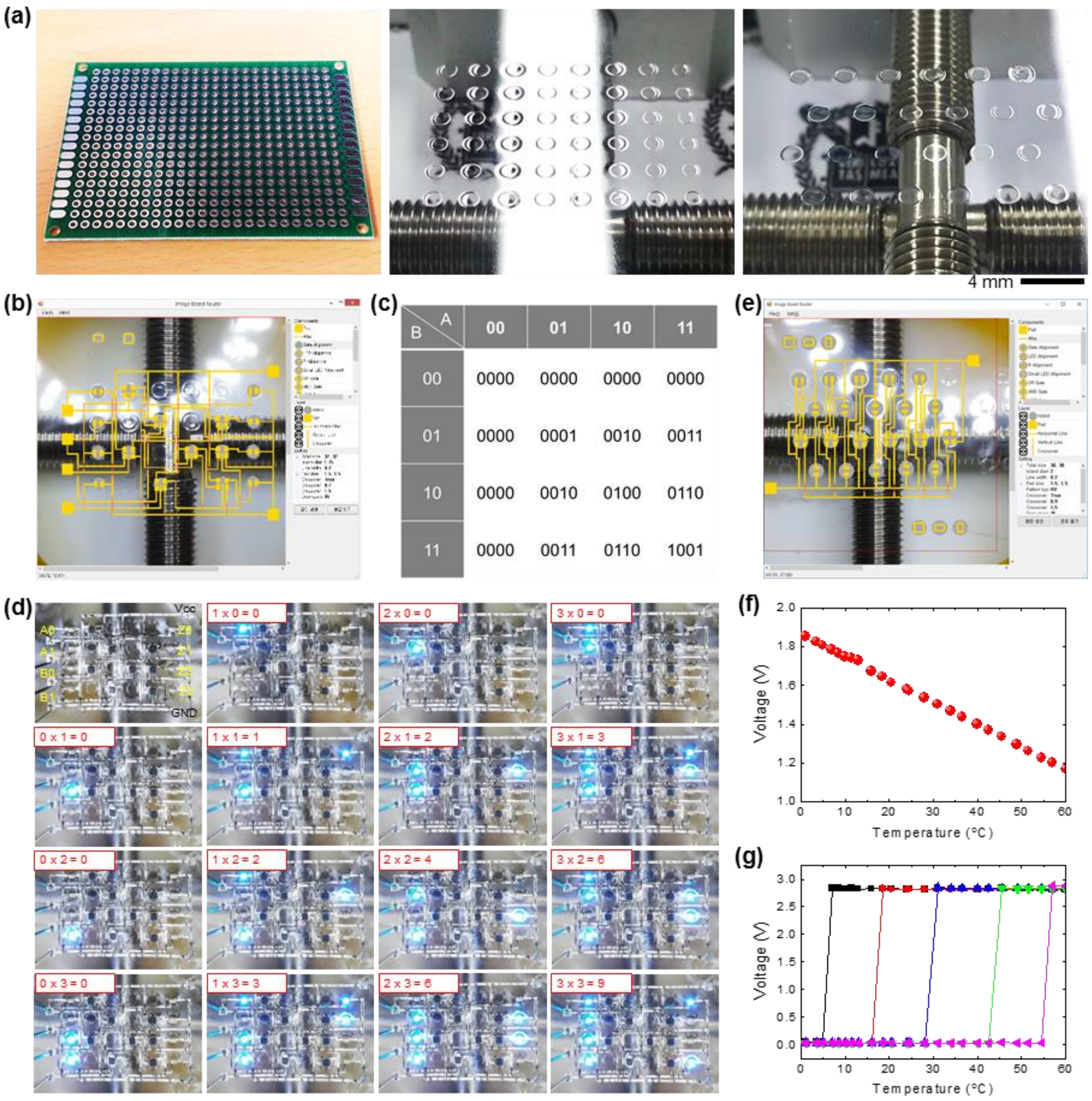

Figure S15. Customized wearable 2-bit multiplier and temperature monitoring system based on universal PRISWs. (a) Photographs of the conventional universal PCB (left) and suggested universal PRISWs containing 6 x 8 (middle) and 4 x 6 PRI arrays (right) with distinct lattice configurations. (b) Screenshot image of the designed 2-bit multiplier circuit layout by the automated circuit networking program. (c) Truth table of 2-bit multiplier. (d) Photographs of full operation of the wearable 2-bit multiplier. (e) Screenshot image of the designed temperature monitoring system circuit layout. (f) Output characteristics of the temperature sensor IC as temperature increases from 0 to 60 ℃. (g) Output characteristics of LEDs integrated in the temperature monitoring system. Each LED is sequentially turned on at 5, 18, 31, 44, 56 ℃.

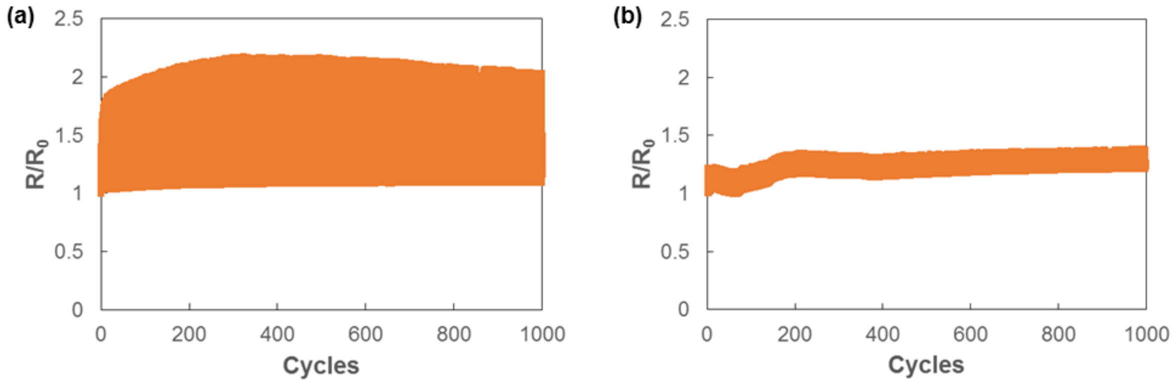

Figure S16. Cyclic reliability of wrinkled interconnects encapsulated by different PDMS. Cyclic reliability of wrinkled interconnect encapsulated by 20:1 PDMS (a) and 30:1 PDMS (b).

Table S1. Comparison between rigid island-based stretchable systems.

Island type	Materials	Process			Thickness [µm]		Skin-conformability	Device-to-island fill factor	Boundary stability	Reference
		Island fabrication	Interconnection wiring	Design freedom	Island	Total platform				
PCB island	PI+PCB	photolithography	photolithography	△	~65	>1000	x	N/A	△	*32*
Protruded island	Composite, PDMS, PET, PI	composite-welding, molding/transfer, photolithography	liquid metal injection, transfer, stencil printing	△	100 ~ 1000	400 ~ 4000	△	0.15 ~ 0.95	△~○	*27*,-*31*
Embedded island	PI, SU8	photolithography	evaporation	△~○	~50	100 ~ 400	△	0.07 ~ 0.23	△~○	*25*, *26*
Embedded island (This work)	PMMA	inkjet-printing	inkjet-printing	◎	7 ~ 15	~50	○	~ 0.45	○	N/A

Stretchable conductor	Fabrication process		Strain absorbing design	Conductivity [S/cm]	Stretchability [%]	Cyclic property	Spatial efficiency	Refere nce
	Method	Design freedom						
Elastic conductor	printing	△~○	N/A	9.7 ~ 738	29 ~ 215	x ~△	○	14, 35-37
Nano-materials	deposition, solution process	△	N/A	1670 ~ 5710	50 ~ 200	△~○	○	38-40
Serpentine trace	photolithography, transfer method	△	in-plane serpentine structure	Deposited metal	40 ~ 300	○	△	43, 44
Nano-/micro-structure	template molding, photolithography	△	nano-/micro-structure	Deposited metal	50 ~ 80	△~○	○	45, 46
This work	prestrain + inkjet printing	◎	vertical wavy structure	~90900	~50	○	○	N/A

Table S2. Recent progress in stretchable conductors.
